# Validity and reliability of the Malay Short Version of the Questionnaire of Olfactory Disorders-Negative Statements (sQOD-NS)

**DOI:** 10.1186/s43163-022-00265-3

**Published:** 2022-06-08

**Authors:** Indumathi Ainer, Salina Binti Husain, Aneeza Khairiyah, Farah Dayana Zahedi, Jegan Thanabalan

**Affiliations:** 1 Department of Otorhinolaryngology, UKM Medical Centre, Jalan Yaacob Latiff, Bandar Tun Razak, 56000 Cheras, Kuala Lumpur, Malaysia; 2 Department of Surgery, Division Neurosurgery, Universiti Kebangsaan Malaysia, Kuala Lumpur, Malaysia; 3 Faculty of Medicine, Universiti Kebangsaan Malaysia, Kuala Lumpur, Malaysia

**Keywords:** Sniffin’ sticks, Olfaction, Questionnaire, Malay version

## Abstract

**Background:**

Olfaction plays an enormous role in every aspect of life including health, emotions, social life, and safety. This is why olfactory dysfunction will leave a great impact on a person’s life. During the recent pandemic hit of coronavirus 2019 (COVID-19), much importance was given to olfaction and its sequelae post-COVID-19. There is various questionnaire being used for determining olfactory disorders worldwide. In Malaysia, Malay language is widely conversed among the local population hence an assessment tool for an olfactory specific quality of life is necessary.

**Method:**

This is a cross-sectional study performed in the Otorhinolaryngology clinic in a tertiary hospital. The short Questionnaire of Olfactory Disorders-Negative Statements (sQOD-NS) is a validated questionnaire that is a simple and easy tool to assess the impact of olfactory dysfunction in daily life. The original version of the short Questionnaire of Olfactory Disorders-Negative Statements (sQOD-NS) questionnaire was translated into the Malay language. A forward-backwards translation, validity, and reliability study was done on this questionnaire. A Malay version of sQOD-NS was responded to by a total of 70 participants including 35 patients of normosia and 35 patients with smell dysfunction and repeated after 2 weeks via phone call response. The discriminant validity, internal consistency, and test re-test reliability were assessed.

**Results:**

The pilot study revealed that participants who were normosmic had a higher mean score than smell dysfunction. A Student *t* test shows mean 20.5 ± 1.22 in normosmic group and 6.06 ± 2.41 in hyposmic group which are significant with *p* value of < 0.01. The coefficient of correlation (*r*) between test and retest scores was 0.77 (*P* < 0.001).

**Conclusion:**

The Malay translation of sQOD-NS is a validated questionnaire that can be used both in clinical practice and in academics.

**Supplementary Information:**

The online version contains supplementary material available at 10.1186/s43163-022-00265-3.

## Introduction

The olfactory is a component of a multi-sensory system. While a person eats and drinks, a complex sensory pathway includes gustation, trigeminal, and olfactory systems. Olfaction picks up various odors from the surrounding via the nostrils. During consumption, food is tasted and smelt at the throat, deciding the final taste. Patients usually complain of bland taste in food after losing their sense of smell.

Olfaction is an important sense used in daily life. Olfaction plays a big role in tasting food and avoiding harmful substances like fire and eating spoilt food [1]. Therefore, olfactory dysfunction not only reduces the quality of life but can also be life-threatening [2].

In relation to the Questionnaire of Olfactory Disorders-Negative Statements (QOD-NS), many studies have been conducted on novel coronavirus-19 (COVID-19) where patients frequently complain of ageusia and anosmia with relation to psychosocial aspects. Recently, Mattos et al. developed a short version of QOD-NS (sQOD-NS) composed of 7 items, with excellent validity and reliability properties [3].

sQOD-NS patient-reported outcome questionnaire including social, eating, annoyance, and anxiety questions. Question 1—change in smell affects social outings, 2—impact on daily social activities, 3—problems with my sense of smell make me more irritable, 4 and 5—smell associated with eating habits. 6 and 7—for anxiety related to change in smell. Items are rated on a scale of 0–3, with higher scores reflecting the better olfactory-specific quality of life (QOL). The total score ranges from 0 (severe impact on QoL) to 21 (no impact on QoL). The item and total scores of sQOD-NS significantly differ between patients with presumed anosmia at the time of the assessment, and those with presumed hyposmia or without olfactory dysfunction (*p = 0.001) (Table 1).

**Table 1:** Short version of Questionnaire of Olfactory Disorders-Negative Statements of patient

Short version QOD-NS items	0	1	2	3
1. Changes in my sense of smell isolate me socially.				
2. The problems with my sense of smell have a negative impact on my daily social activities				
3. The problems with my sense of smell make me more irritable				
4.Because of the problems with my sense of smell, I eat out less				
5.Because of the problems with my sense of smell, I eat less than before (loss of appetite)				
6. Because of the problems with my sense of smell, I have to make more effort to relax				
7. I'm afraid I'll never be able to get used to the problems with my sense of smell.				
**Short version QOD-NOS score**				
Total score				

Malaysia is well known for its multi-ethnicity and using the Malay language as a universal communication tool at least among locals. In order to assist locals to understand better a translated version of sQOD-NS was proposed in this study. The study aims to easily administer this questionnaire and obtain valid answers from the local population. This is to provide a validated tool to assess psychosocial outcomes in olfactory dysfunction among the Malaysian population in the future. To date, sQOD-NS is only validated in English, Spanish, and French [4]. There is no validated sQOD-NS version for Malay-speaking countries that include more than 30 million populations.

## Material and methods

### Participants

This was a cross-sectional study carried out in the outpatient department of Otorhinolaryngology clinic, Universiti Kebangsaan Malaysia Medical Centre (UKMMC), from February 2021 till October 2021. Ethics approval was obtained from the UKMMC ethics committee ((FF-2021-116C).

Two groups of patients were recruited which were patients with normosmic and hypo-anosmic. The subjects with a reduced sense of smell were recruited from patients attending the otorhinolaryngology clinic. Exclusion criteria were subjects less than 18 years old, pregnant patients, an underlying neurodegenerative disorder, for example, Alzheimer’s disease and Parkinson’s disease, neuropsychiatric disorder, and upper respiratory tract infection within 2 weeks. Healthy subjects with a normal sense of smell were invited to participate in the study voluntarily among the hospital staff and medical students.

### Translation procedure

Permission was obtained from the original author of sQOD-NS through email for translation and validation of the questionnaire. Two experienced clinicians and non-medical professionals evaluated the original sQOD-NS and assessed the relevance of each question. For each question, the forward translation from English to Malay was done and was evaluated on the relevance to the local language. The two forward translations will be reviewed and reconciled by the research team’s four members who are bilingual with good command in both languages. The reconciliation of translations will strive for consensus among research team members in producing a preliminary forward translation version of the sQOD-NS.

A backward translation was done by another two bilingual professionals. The backward translated questionnaire was assessed to ensure the original meaning remained. The translation was made sure not done word by word but in phrases so the original meaning was understood and not changed. A final version of the forward-backwards translated questionnaire was then ready for validation. A comparison of the original sQOD-NS and translated version are available.

### Content validity

A panel of four otorhinolaryngologists re-assessed the Malay version of sQOD-NS questionnaire. The panelist commented on (1) the clarity of the wording of questions (appropriateness); and (2) appropriateness for local language usage.

### Face validity

The Malay version of sOQD-NS was then pre-tested among 30 normosmic patients. These patients were not included in the validation study later. They evaluated all the items in the Malay version of sOQD-NS, whether all sentences and options were clear and easily understood. Some items were reworded based on the comments from these participants. The final Malay version of sQOD-NS is concluded.

### Discriminant validity, internal consistency, and test re-test reliability

The final Malay version of sQOD-NS was then answered by a total of 70 participants, including 35 hypo-anosmic patients with AR and 35 normosmic controls. Both groups were invited to answer the questionnaire. The participants repeated responses to the questionnaire after a 2-week interval via phone. This was done to assess for discriminant validity, internal consistency, and test re-test reliability of the Malay version of sQOD-NS questionnaire.

### Statistical analysis

The calculation of sample size for the validity of the Malay version of sQOD-NS questionnaire was based on 5 subjects per item for each arm, a total of 10 subjects per item. Discriminant validity was evaluated by comparing the mean ± standard deviation (SD) of total sQOD-NS between two groups by *t* test. Also, the score for each item in the questionnaire was compared between groups using chi-square or Kendall’s tau-b test. The internal consistency of the questionnaire and items was determined using the Cronbach alpha, a value of 0.5–0.7 was acceptable and 0.7–0.8 was good [5].

The test-retest reliability of the total Malay version of sQOD-NS score was assessed by intraclass correlation coefficient. An ICC value between 0.5 and 0.75 indicated moderate reliability, values between 0.75 and 0.9 indicated good reliability, and values larger than 0.9 indicated excellent reliability [6]. The data is presented as a proportion (95% confidence interval).

## Results

### Participants

The characteristic of both normosmic and hypo-anosmic groups is presented in Table 2. There was no difference in gender, age, and race.

**Table 2:** Summary of demographic data according to age, gender, and ethnicity

	Normosmia	Hyposmia	*P*
*N*	35	35	
Gender			
% female	19/35	16/35	0.83
Mean age	38.4	41.97	
Age group			
% < 20	2.9	0	0.90
% 21–30	25.7	14.3	
%31–40	31.4	34.3	
% 41–50	20	25.7	
% 51–60	14.3	14.3	
%> 60	5.7	11.4	
Race			
% Malay	45.7	40	0.08
% Chinese	40	42.9	
% India	14.3	14.3	
% Others	0	2.9	

### Content validity

A panel that was involved in the content validation process was given a total score of 10 (minimum 0 and maximum 10) weighing the points (1) the clarity of the wording of questions, and (2) appropriateness for local language usage. Then the score was calculated. The content validity index (CVI) was calculated based on the total scores rated by each expert (Table 3).

**Table 3:** Content validity index of Malay version of sQOD-NS

Experts	1	2	3	4	5	6	7	Total
Rater 1	10/10	10/10	8/10	10/10	8/10	10/10	10/10	66/70
Rater 2	10/10	10/10	8/10	8/10	10/10	10/10	10/10	66/70
Rater 3	10/10	10/10	8/10	8/10	8/10	10/10	10/10	64/70
Rater 4	10/10	10/10	8/10	10/10	8/10	10/10	8/10	64/70
Score								260/280
Content validity index								0.93

### Discriminant validity, internal consistency, and test re-test reliability

The total Malay version of sQOD-NS score showed good discrimination validity between AR and healthy controls (13.44 ± 1.58 v 1.00 ± 2.12, *p* < 0.01) which was also reflected in each item (Table 4). The Malay version of sQOD-NS showed good internal consistency with Cronbach’s alpha of 0.73 (95% CI 0.90–0.94). The corrected item-total correlation values for each item were more than 0.3, indicating that each item correlates well with the total score (Table 5). The intraclass correlation coefficient of total the Malay version of sQOD-NS was 0.77 (95% CI 0.56–0.87) with *p* < 0.01, indicating a good test-retest reliability (Table 6).

**Table 4:** Comparison of the Malay version of sqOD-NS scores between smell dysfunction patients and healthy control

First test	Normosmia	Hyposmia	*P*
*N*	35	35	
Total sQOD-NS Mean (SD)	20.5 ± 1.22	6.06 ± 2.41	< 0.01
Question 1			
Score of 0 (%)	0	11.4	
Score of 1 (%)	0	88.6	
Score of 2 (%)	8.6	0	
Score of 3 (%)	91.4	0	
Question 2			
Score of 0 (%)	0	20	<0.01
Score of 1 (%)	0	60	
Score of 2 (%)	11.4	20	
Score of 3 (%)	88.6	0	
Question 3			
Score of 0 (%)	0	31.4	< 0.01
Score of 1 (%)	0	60	
Score of 2 (%)	11.4	3	
Score of 3 (%)	88.6	0	
Question 4			
Score of 0 (%)	0	22.9	< 0.01
Score of 1 (%)	0	74.3	
Score of 2 (%)	8.6	2.9	
Score of 3 (%)	91.4	0	
Question 5			
Score of 0 (%)	0	37.1	< 0.01
Score of 1 (%)	0	57.1	
Score of 2 (%)	5.7	2	
Score of 3 (%)	94.3	0	
Question 6			
Score of 0 (%)	0	17.1	< 0.01
Score of 1 (%)	0	68.6	
Score of 2 (%)	2.9	14.3	
Score of 3 (%)	97.1	0	
Question 7			
Score of 0 (%)	0	34.3	< 0.01
Score of 1 (%)	0	65.7	
Score of 2 (%)	2.9	0	
Score of 3 (%)	97.1	0

**Table 5:** Internal consistency of sQOD-NS

Item	Corrected item-total correlation	Cronbach’s α if item deleted	Cronbach’s α
1	0.492	0.705	0.734
2	0.585	0.664	
3	0.446	0.703	
4	0.479	0.695	
5	0.398	0.714	
6	0.432	0.705	
7	0.360	0.720

**Table 6:** Reliability result of each item in Malay version of sQOD-NS

Item	Intraclass correlation (lower bound, upper bound) 95% CI	SE	*P*
Total sQOD-NS	0.77 (0.51, 0.87)		< 0.01 ICC (2 way mixed, absolute agreement)
Question 1	0.77 (0.55, 0.89)		< 0.01 (ICC)
Question 2	0.96 (0.92, 0.98)		< 0.01
Question 3	0.98 (0.96, 0.99)		< 0.01
	0.95 (0.85,0.99)	0.05	< 0.01 (Cohen)
Question 4	0.69 (0.40, 0.84)		< 0.01
Question 5	0.74 (0.50, 0.87)		< 0.01 (ICC)
Question 6	0.69 (0.38, 0.84)		< 0.01
Question 7	0.86 (0.71, 0.93)		< 0.01

## Discussion

The Malay version of sQOD-NS questionnaire was originally in the English language. This questionnaire was rebuilt to suit the Malaysian population in terms of familiarity. A self-administered questionnaire like sQOD-NS provides an easier method of screening and determining olfactory dysfunction and its social impact among the Malaysian population. The original version of sQOD-NS was established in 2019 and validated. The sQOD-NS questionnaire has been used in many studies for chronic rhinosinusitis and COVID-19 [7]. For utilization in the Malaysian population, a cross-cultural translation and adaptation are vital for better comprehension of the study population and further studies on olfactory dysfunction can be performed. The Malay version of sQOD-NS can be employed by physicians to identify patients with smell disturbances in an outpatient setting.

The most imperative building of this questionnaire is to identify smell related quality of life impact in a targeted population. A forward and backward translation was performed on the original version by two groups of people who were fluent in both Malay and English languages. A medical group will help to recognize the concept and objective of the questionnaire; while non-medical professionals were to help avoid medical jargon administered in the questionnaire.

Content validity is indispensable as it displays that the questionnaire is fully representative of the aim. Content validity index (CVI) calculated from a scoring scale given to a panel of experts for a quantitative measure. A scale of more than 0.9 indicates excellent CVI. The CVI in this study is 0.93. A previous study that adapted the QOD-NS in the French population showed a CVI of 0.9 [8].

The present study shows good reliability evident by a Cronbach’s alpha of 0.73. This is comparable to a previous study using sQOD-ND which has Cronbach’s alpha of 0.80 [8].

It indicates that the Malay version of sQOD-NS has a good internal consistency in detecting olfactory dysfunction even after the translation procedure. Items that had lower corrected item-total correlation were re-evaluated. To date, there is no specific criteria or cut off point to eliminate or retain an item within the questionnaire. Studies had adopted that corrected item—total correlation with the value of greater than 0.3—should be retained [9]. Item 7 was retained in the questionnaire as this is self-administered. No further amendments were made to the participant’s response.

Test-retest reliability for the total Malay version of sQOD-NS score in this present study shows a value of 0.77. This is comparable with other studies showing an *r* value of 0.83 and 0.8612 [13]. Cohen kappa coefficient (*k*) of all items in the Malay version of sQOD-NS are good with the value ranging from 0.7 to 1.0 (*p* < 0.01)

Sniffin’ stick test was first invented by Hummel in 1997 [10]. Since then it has been validated in many countries. The advantage is to provide a comprehensive assessment by including a threshold test, odor discrimination test, and identification test. Sniffin’s stick result was used as a criterion in the present study to define the patients with normosmia and hypo-anosmia individuals. The Malay version of sQOD-NS has an acceptable discriminant validity by comparing the normosmia group and hypo-anosmia group.

## Conclusion

The Malay version of sQOD-NS is a valid and reliable questionnaire that is suitable for use in Malaysia. It may be used as an easy and self-administered screening tool in population studies to identify patients with olfactory dysfunction.

The main limitation of this study is the low number of patients. Some items of sQOD-NS involved the social habits of individuals. During the pandemic, Malaysian governments have imposed lockdown in some regions, modifying the social habits of populations, which may impact the patient responses. Other than that, only the Malay version of this modified questionnaire was validated and those not literate in Bahasa Malaysia will have difficulty answering this self-administered questionnaire. The strength of this study is the realization of psychophysical olfactory evaluations, allowing the confirmation of olfactory dysfunction.

## Supplementary Information

**Additional file 1.**

## Data Availability

All data generated or analysed during this study are included in this published article and its supplementary information files.
