# Supplementary material for: Validity and reliability of the Malay Short Version of the Questionnaire of Olfactory Disorders-Negative Statements (sQOD-NS)
Source: Egypt J Otolaryngol. 2022 Jun 8;38(1):74. doi: 10.1186/s43163-022-00265-3 (PMC9176160; doi:10.1186/s43163-022-00265-3)
Supplement: Supplementary file 1 — Additional file 1. [file 43163_2022_265_MOESM1_ESM.docx]

Appendix

Table 1: Short version of Questionnaire of Olfactory Disorders-Negative Statements of patient.

| **Short version QOD-NS items** | 0 | 1 | 2 | 3 |
| --- | --- | --- | --- | --- |
| 1. Changes in my sense of smell isolate me socially. |  |  |  |  |
| 2. The problems with my sense of smell have a negative impact on my daily social activities |  |  |  |  |
| 3. The problems with my sense of smell make me more irritable |  |  |  |  |
| 4.Because of the problems with my sense of smell, I eat out less |  |  |  |  |
| 5.Because of the problems with my sense of smell, I eat less than before (loss of appetite) |  |  |  |  |
| 6. Because of the problems with my sense of smell, I have to make more effort to relax |  |  |  |  |
| 7. I'm afraid I'll never be able to get used to the problems with my sense of smell. |  |  |  |  |
| **Short version QOD-NOS score** |  |  |  |  |
| Total score | | | |  |

Table 2: Versi pendek Soal Selidik Olfactory Disorders-Negative Statements pesakit.

| Item versi pendek QOD-NS | Dianggap anosmik | Dianggap hiposmik | Tiada kehilangan bau |
| --- | --- | --- | --- |
| 1. Perubahan deria bau mengasingkan saya secara sosial. |  |  |  |
| 1. Masalah dengan deria bau saya memberi kesan negative   kepada aktiviti sosial harian saya |  |  |  |
| 1. Masalah dengan deria bau saya menjadikan saya lebih mudah marah |  |  |  |
| 1. Oleh kerana masalah dengan deria bau saya, saya kurang makan |  |  |  |
| 1. Kerana masalah dengan deria bau saya,   saya makan lebih sedikit daripada sebelumnya (hilang selera makan) |  |  |  |
| 1. Oleh kerana masalah dengan deria penciuman saya,   saya harus berusaha lebih kuat untuk berehat |  |  |  |
| 1. Saya takut saya tidak akan pernah dapat membiasakan diri   dengan masalah deria bau saya. |  |  |  |
| Skor keseluruhan versi pendek QOD-NOS |  |  |  |

Table 3: Malay version of sQOD-NS

| Item versi pendek QOD-NS | 0 | 1 | 2 | 3 |
| --- | --- | --- | --- | --- |
| 1. Perubahan deria bau mengasingkan saya secara sosial. |  |  |  |  |
| 1. Masalah dengan deria bau saya memberi kesan negatif kepada aktiviti sosial harian saya |  |  |  |  |
| 1. Masalah dengan deria bau menjadikan saya lebih mudah marah |  |  |  |  |
| 1. Oleh kerana masalah dengan deria bau, saya kurang makan |  |  |  |  |
| 1. Kerana masalah dengan deria bau saya, saya hilang selera makan |  |  |  |  |
| 1. Oleh kerana masalah dengan deria bau, saya harus berusaha lebih kuat untuk berehat |  |  |  |  |
| 1. Saya takut saya tidak akan pernah dapat membiasakan diri dengan masalah deria bau saya. |  |  |  |  |
| Skor keseluruhan versi pendek QOD-NOS |  |  |  |  |

Table 4: Summary of demographic data according to age, gender and ethnicity

|  | Normosmia | Hyposmia | P |
| --- | --- | --- | --- |
| N | 35 | 35 |  |
| Gender  % female | 19/35 | 16/35 | 0.83 |
| Mean age | 38.4 | 41.97 |  |
| Age group  % <20  % 21-30  %31-40  % 41-50  % 51-60  % >60 | 2.9  25.7  31.4  20  14.3  5.7 | 0  14.3  34.3  25.7  14.3  11.4 | 0.90 |
| Race  % Malay  % Chinese  % India  % Others | 45.7  40  14.3  0 | 40  42.9  14.3  2.9 | 0.08 |

Table 5: Content Validity Index of Malay version of sQOD-NS

| Experts | 1 | 2 | 3 | 4 | 5 | 6 | 7 | Total |
| --- | --- | --- | --- | --- | --- | --- | --- | --- |
| Rater 1 | 10/10 | 10/10 | 8/10 | 10/10 | 8/10 | 10/10 | 10/10 | 66/70 |
| Rater 2 | 10/10 | 10/10 | 8/10 | 8/10 | 10/10 | 10/10 | 10/10 | 66/70 |
| Rater 3 | 10/10 | 10/10 | 8/10 | 8/10 | 8/10 | 10/10 | 10/10 | 64/70 |
| Rater 4 | 10/10 | 10/10 | 8/10 | 10/10 | 8/10 | 10/10 | 8/10 | 64/70 |
| Score | | | | | | | | 260/280 |
| Content Validity Index | | | | | | | | 0.93 |

Table 6: Comparison of the Malay version of sqOD-NS scores between smell dysfunction patients and healthy control

| First test | Normosmia | Hyposmia | P |
| --- | --- | --- | --- |
| N | 35 | 35 |  |
| Total sQOD-NS Mean (SD) | 20.5 ± 1.22 | 6.06 ±2.41 | <0.01 |
| Question 1  Score of 0 (%)  Score of 1 (%)  Score of 2 (%)  Score of 3 (%) | 0  0  8.6  91.4 | 11.4  88.6  0  0 |  |
| Question 2  Score of 0 (%)  Score of 1 (%)  Score of 2 (%)  Score of 3 (%) | 0  0  11.4  88.6 | 20  60  20  0 | <0.01 |
| Question 3  Score of 0 (%)  Score of 1 (%)  Score of 2 (%)  Score of 3 (%) | 0  0  11.4  88.6 | 31.4  60  3  0 | <0.01 |
| Question 4  Score of 0 (%)  Score of 1 (%)  Score of 2 (%)  Score of 3 (%) | 0  0  8.6  91.4 | 22.9  74.3  2.9  0 | <0.01 |
| Question 5  Score of 0 (%)  Score of 1 (%)  Score of 2 (%)  Score of 3 (%) | 0  0  5.7  94.3 | 37.1  57.1  2  0 | <0.01 |
| Question 6  Score of 0 (%)  Score of 1 (%)  Score of 2 (%)  Score of 3 (%) | 0  0  2.9  97.1 | 17.1  68.6  14.3  0 | <0.01 |
| Question 7  Score of 0 (%)  Score of 1 (%)  Score of 2 (%)  Score of 3 (%) | 0  0  2.9  97.1 | 34.3  65.7  0  0 | <0.01 |

Table 7: Internal consistency of sQOD-NS

| Item | Corrected Item- Total Correlation | Cronbach’s α if Item Deleted | Cronbach’s α |
| --- | --- | --- | --- |
| 1 | 0.492 | 0.705 | 0.734 |
| 2 | 0.585 | 0.664 |  |
| 3 | 0.446 | 0.703 |  |
| 4 | 0.479 | 0.695 |  |
| 5 | 0.398 | 0.714 |  |
| 6 | 0.432 | 0.705 |  |
| 7 | 0.360 | 0.720 |  |

Table 8: Reliability result of each item in Malay version of sQOD-NS

| Item | Intraclass correlation (Lower Bound, Upper Bound) 95% CI | SE | P |
| --- | --- | --- | --- |
| Total sQOD-NS | 0.77(0.51, 0.87) |  | <0.01  ICC (2 way mixed, absolute agreement) |
| Question 1 | 0.77 (0.55, 0.89) |  | <0.01 (ICC) |
| Question 2 | 0.96 (0.92, 0.98) |  | < 0.01 |
| Question 3 | 0.98 (0.96, 0.99)  0.95 (0.85,0.99) | 0.05 | < 0.01  < 0.01 (Cohen) |
| Question 4 | 0.69 (0.40, 0.84) |  | < 0.01 |
| Question 5 | 0.74 (0.50,0.87) |  | < 0.01 (ICC) |
| Question 6 | 0.69 (0.38, 0.84) |  | <0.01 |
| Question 7 | 0.86 (0.71,0.93) |  | <0.01 |
